# The intestinal TORC2 signaling pathway contributes to associative learning in *Caenorhabditis elegans*

**DOI:** 10.1371/journal.pone.0177900

**Published:** 2017-05-25

**Authors:** Naoko Sakai, Hayao Ohno, Masahiro Tomioka, Yuichi Iino

**Affiliations:** 1 Department of Biological Sciences, Graduate School of Science, University of Tokyo, Tokyo, Japan; 2 Molecular Genetics Research Laboratory, Graduate School of Science, University of Tokyo, Tokyo, Japan; University of California San Francisco, UNITED STATES

## Abstract

Several types of associative learning are dependent upon the presence or absence of food, and are crucial for the survival of most animals. Target of rapamycin (TOR), a kinase which exists as a component of two complexes, TOR complex 1 (TORC1) and TOR complex 2 (TORC2), is known to act as a nutrient sensor in numerous organisms. However, the *in vivo* roles of TOR signaling in the nervous system remain largely unclear, partly because its multifunctionality and requirement for survival make it difficult to investigate. Here, using pharmacological inhibitors and genetic analyses, we show that TORC1 and TORC2 contribute to associative learning between salt and food availability in the nematode *Caenorhabditis elegans* in a process called taste associative learning. Worms migrate to salt concentrations experienced previously during feeding, but they avoid salt concentrations experienced under starvation conditions. Administration of the TOR inhibitor rapamycin causes a behavioral defect after starvation conditioning. Worms lacking either RICT-1 or SINH-1, two TORC2 components, show defects in migration to high salt levels after learning under both fed and starved conditions. We also analyzed the behavioral phenotypes of mutants of the putative TORC1 substrate RSKS-1 (the *C*. elegans homolog of the mammalian S6 kinase S6K) and the putative TORC2 substrates SGK-1 and PKC-2 (homologs of the serum and glucocorticoid-induced kinase 1, SGK1, and protein kinase C-α, PKC-α, respectively) and found that neuronal RSKS-1 and PKC-2, as well as intestinal SGK-1, are involved in taste associative learning. Our findings shed light on the functions of TOR signaling in behavioral plasticity and provide insight into the mechanisms by which information sensed in the intestine affects the nervous system to modulate food-searching behaviors.

## Introduction

Target of rapamycin (TOR) is a serine/threonine kinase conserved from yeast to humans that performs many diverse functions by forming two structurally distinct protein complexes: TOR complex 1 (TORC1) and TOR complex 2 (TORC2) [1–3]. The use of rapamycin—a potent TORC1 inhibitor—has enabled considerable advancements in understanding the functions of the TORC1 signaling pathway, and TORC1 is now widely recognized for its role in monitoring nutrient information and regulating gene expression and metabolism, thereby controlling cell growth [4]. By contrast, assessing TORC2 function has been challenging due to a lack of specific inhibitors.

TORC2 contains four core components that are all highly conserved across species: rapamycin-insensitive companion of TOR (Rictor), stress-activated protein kinase-interacting protein (Sin1), lethal with SEC13 protein 8 (LST8), and TOR. Previous studies have identified certain AGC-subfamily kinases as putative TORC2 substrates, including Akt, protein kinase C-α (PKC-α), and serum and glucocorticoid-induced kinase 1 (SGK1), and TORC2 has been shown to control the actin cytoskeleton as well as gene transcription through phosphorylation of its substrates [5,6]. Furthermore, TORC2 has also been reported to regulate neuronal morphology, long-term memory formation, and chronic opiate-induced changes in neuronal excitation [7,8]. However, evaluation of the neuronal functions of TORC2 *in vivo* remains challenging because loss of TORC2 is lethal in many species. The nematode *Caenorhabditis elegans* is notable in this regard in that viable mutants of *rict-1*, the sole ortholog of Rictor, have been reported [9,10].

*C*. *elegans* has been widely used as a model organism in neuroscience research because it demonstrates various types of complex behaviors despite its morphological simplicity. Previously, we demonstrated that worms can exhibit a form of associative learning that depends on food availability and external salt concentration [11,12]: when worms were cultivated on a medium containing NaCl and bacterial food, they learned to migrate to the NaCl concentration at which they had been previously grown. Conversely, after exposure to NaCl in the absence of food, the worms learned to avoid the previously experienced NaCl concentration (S1 Fig). We call this salt and feeding state dependent behavioral plasticity “taste associative learning.” We have reported that certain evolutionarily conserved signaling pathways, including the insulin/PI3-kinase (PI3K) and the Gq/nPKC pathways, are required for taste associative learning [12,13]. Mutants of the insulin/PI3K pathway show defects in avoidance of NaCl concentrations after conditioning in the absence of food, although they show normal attraction to NaCl concentrations experienced during feeding [11]. After starvation at a high salt concentration, these mutants migrated towards high salt concentrations instead of low salt concentrations, in a pattern similar to worms that were previously fed at a high salt concentration. A mirror-image behavior, *i*.*e*. migration to low salt, was observed after starvation at a low salt concentration. These observations suggest that the insulin/PI3K pathway is required for associative learning between NaCl concentration and starvation, and/or learning-dependent switching from attraction to avoidance of a previously experienced salt concentration. On the other hand, increased activity of the Gq/PKC pathway promotes high salt migration irrespective of conditioning [11]. In particular, after either feeding at a low salt concentration or starvation at a high salt concentration, mutants with increased activity of the pathway migrated to high salt concentrations instead of low. These observations suggested that this pathway plays instructive roles in promoting high-salt migration, acting either at the memory acquisition step or during the chemotaxis behavior. So far it is not known at exactly which step of the learning and behavioral execution processes that these pathways actually regulate taste associative learning.

In this study, we aimed to identify new players in taste associative learning. By performing experiments in which we used pharmacological inhibitors and genetic mutants, we determined that disruption of the *C*. *elegans* TORC1 (CeTORC1) and TORC2 (CeTORC2) pathways resulted in abnormal behaviors in taste associative learning. We showed that RSKS-1, the homologue of S6 kinase (S6K), which acts downstream of TORC1 [4], functions in neurons. Moreover, we found that the putative CeTORC2 substrates PKC-2 and SGK-1 control taste associative learning by acting in distinct locations, in neurons and the intestine, respectively, and further that SGK-1 functions downstream of TORC2. Our results raise the possibility that the chemical information sensed in the intestine affects the nervous systems to modulate this food-related behavior in *C*. *elegans*.

## Results

### Loss of CeTORC1 signaling disrupts normal taste associative learning

**Fig 1:**
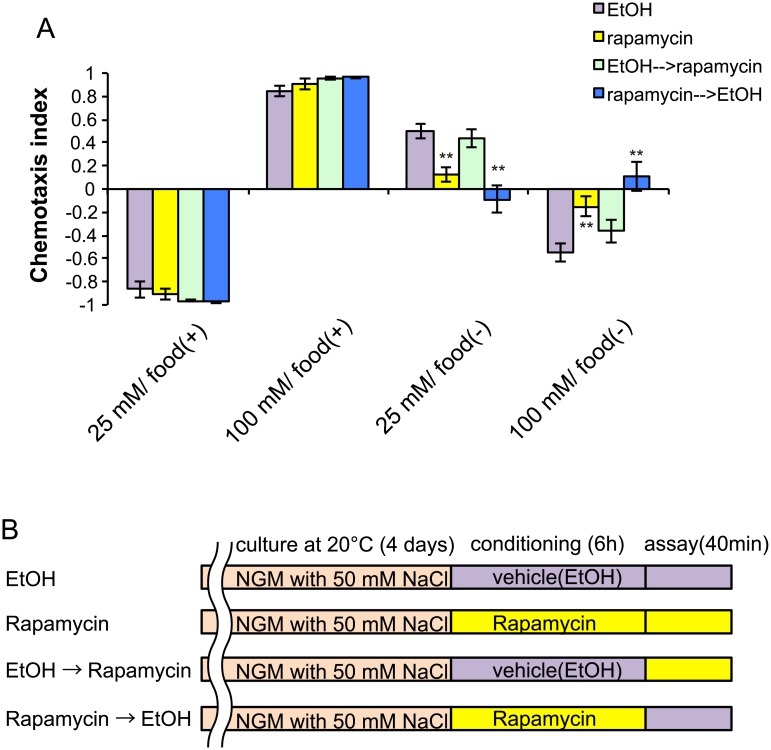
TOR-specific inhibitor rapamycin disrupts normal taste associative learning. Chemotaxis indices of wild-type worms after conditioning on low (25 mM) or high (100 mM) salt-containing agar plates with (+) or without (–) food. (B) Schematic representation of the timing of rapamycin treatment. Rapamycin or vehicle was administered to worms after 4 days of cultivation. Error bars, s.e.m.; **p < 0.01 (EtOH vs. each condition, one-way ANOVA with Dunnett’s post hoc test, N ≥ 4).

To examine the role of TOR signaling in taste associative learning, we inhibited TOR activity by using rapamycin. Rapamycin has been used as a TOR inhibitor in several organisms, and it is also effective in *C*. *elegans* at high concentrations (100 μM) [14]. We administered 100 μM rapamycin or vehicle to worms during both salt conditioning and the salt-chemotaxis test, and found that the treatment weakened the avoidance of the salt concentrations at which worms were starved: rapamycin-treated animals showed decreased migration to high or low salt levels after exposure to 25 or 100 mM NaCl in the absence of food, respectively, while chemotaxis after feeding was not affected (Fig 1A); these findings imply that post-developmental inhibition of TOR signaling may result in defective taste associative learning during starved conditions. We treated worms with rapamycin during either salt conditioning or salt-chemotaxis testing (Fig 1B). Rapamycin administration during chemotaxis testing was not sufficient to disrupt taste associative learning, whereas rapamycin exposure during salt conditioning disrupted normal chemotaxis behavior (Fig 1A).

**Fig 2:**
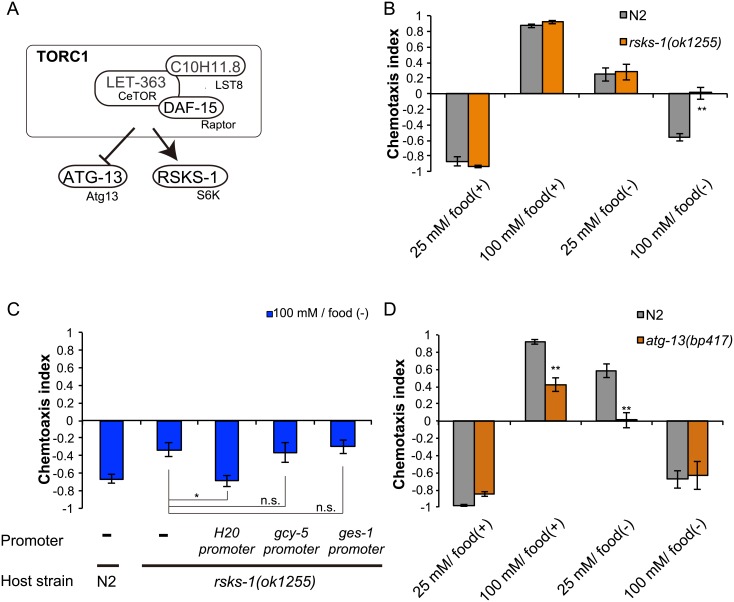
TORC1 signaling regulates migration to low salt concentrations through S6K. (A) A schematic diagram of putative TORC1 signaling in *C*. *elegans*. (B) Salt chemotaxis of wild-type N2 worms and *rsks-1(ok1255)* mutants after salt conditioning. (C) Expression of *rsks-1* cDNA under the pan-neuronal *H20* promoter, but not ASER-specific *gcy-5* promoter or intestinal *ges-1* promoter, rescued the chemotaxis abnormality of *rsks-1(ok1255)* mutants. Salt chemotaxis was tested after high-salt/food(–) conditioning. (D) Salt chemotaxis of wild-type N2 animals and *atg-13(bp417)* mutants after salt conditioning. *p < 0.05, **p < 0.01, n.s. = not significant (one-way ANOVA with Dunnett’s post hoc test, N ≥ 9).

In mammalian cells, S6K, eIF4E-binding protein 1 (4E-BP1), and autophagy related gene 13 (Atg13) are major substrates of TORC1 (Fig 2A) [4]. However, null mutants of CeTORC1 core components, *let-363*/TOR, *daf-15*/Raptor, and *C10H11*.*8*/LST8, are all lethal, and no structural 4E-BP1 homolog has been found in the *C*. *elegans* genome [15]. Therefore, in our assay we tested mutants of the putative CeTORC1 targets *rsks-1*, the S6K homolog, and *atg-13*, the Atg13 homolog. Similar to rapamycin-treated animals, *rsks-1* mutants failed to avoid the high salt concentration experienced during starvation: they did not migrate to low salt concentrations after exposure to 100 mM NaCl in the absence of food (high-salt/food(–) conditioning) (Fig 2B, S2 Fig). We investigated the sites of *rsks-1* function by performing tissue-specific rescue experiments, and found that neuronal but not intestinal expression of *rsks-1* cDNA rescued the chemotaxis abnormality (Fig 2C). However, *rsks-1* cDNA expression in only the salt-sensing neuron ASER, which senses chloride ions and is considered to play a primary role in taste associative learning [11,13,16,17], failed to rescue the *rsks-1* defects in taste associative learning. These results suggest that *rsks-1* acts in neurons other than ASER to promote migration to low salt levels after high-salt/starvation conditioning. We also note that *atg-13* mutants migrated to lower salt levels than wild-type animals after high-salt/food(+) and low-salt/food(–) conditioning (Fig 2D). Atg13 is known to be directly phosphorylated and inhibited by TORC1 in mammals [18–20]. Thus, TORC1 might negatively regulate ATG-13 function in salt chemotaxis in *C*. *elegans* (see also Discussion).

### CeTORC2-pathway mutants show marked defects in associative learning

**Fig 3:**
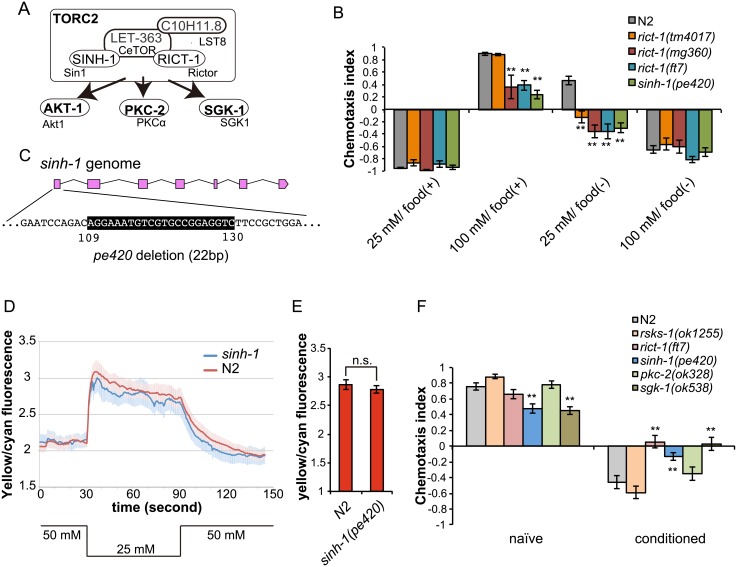
TORC2 components regulate migration to high salt concentrations. (A) A schematic model of putative TORC2 signaling in *C*. *elegans*. (B) Salt chemotaxis of wild-type N2 worms and mutants of TORC2 components. TORC2-signaling mutants migrated to lower salt levels than did wild-type worms. (C) Genomic structure of *sinh-1*. Pink boxes indicate exons. The location of the *pe420* deletion is shown below the *sinh-1* locus. (D) Calcium responses of ASER of wild-type animals and *sinh-1* mutants to an NaCl down-step from 50 to 25 mM and back to 50 mM (traces represent the means, the shadings the s.e.m.) after 5-h low-salt/food(–) conditioning. (E) Bar graphs showing averaged yellow/cyan fluorescence ratios during 10 s after downstep stimulation. Error bars, s.e.m.; n.s. = not significant (Student’s *t* test, N ≥ 9). (F) Benzaldehyde chemotaxis of wild-type N2 and mutant worms after benzaldehyde conditioning or in the naive state. *rict-1(ft7)*, *sinh-1(pe420)*, and *sgk-1(ok538)* mutants, but not *pkc-2(ok328)* and *rsks-1(ok1255)* mutants, showed defects in food-odor associative learning. Error bars, s.e.m.; **p < 0.01, n.s. = not significant (wild-type vs. each mutant, one-way ANOVA with Dunnett’s post hoc test, N ≥ 9).

We note that the phenotype of the *rsks-1* mutant mimics only a part of the rapamycin treated worms’ behavior. Because long-term administration of rapamycin has been reported to be capable of inhibiting TORC2 signaling [14,21], we suspected that in addition to CeTORC1, CeTORC2 might play a role in taste associative learning. As expected, mutants of *rict-1*, which encodes the homolog of the essential TORC2 component Rictor, also showed marked defects in taste associative learning (Fig 3A and 3B, S2 Fig). We tested three previously obtained alleles of *rict-1* and found that the mutant worms migrated to low salt concentrations even after exposure to 25 mM NaCl in the absence of food (low-salt/food(–) conditioning). Previously, the *rict-1(ft7)* nonsense allele and the *rict-1(mg360)* missense allele were reported to cause severe defects in growth and body size and were considered to be loss of function alleles [9,10]. In addition to the chemotaxis defects observed after starvation conditioning, worms harboring these *rict-1* alleles showed decreased migration to high salt concentration after exposure to 100 mM NaCl in the presence of food (high-salt/food(+) conditioning). The *rict-1(tm4017)* mutant showed a defect only after low-salt/food(–) conditioning (Fig 3B). The allele *rict-1(tm4017)* contains a deletion in an intronic region of *rict-1*, and worms carrying this allele showed no obvious defect in growth and body size, which implies that this is a reduction of function allele (S3 Fig). Thus, the differences in the behavioral phenotype might be due to a residual function of *rict-1(tm4017)*. We also tested a mutant of *sinh-1*, which is the homolog of Sin1, another TORC2-specific component [22–24]. Because no *sinh-1* mutant has been reported to date in *C*. *elegans*, we created a putative null *sinh-1(pe420)* mutant by using the CRISPR-Cas9 system (Fig 3C). The *sinh-1(pe420)* mutant worms showed an increase in body fat (S4 Fig), a phenotype similar to that of *rict-1(ft7)* mutants [9]. The results of behavioral assays further showed that *sinh-1* mutants, like the *rict-1* mutants, migrated to lower salt levels than wild-type worms after both high-salt/food(+) and low-salt/food(–) conditioning (Fig 3B, S2 Fig), which agrees with the previous report that Rictor and Sin1 function together in TORC2 [22–24]. Taken together, these data show that TORC2 disruption causes migration to lower salt levels than normal as a result of impairment of either the associative learning or execution of memory-dependent chemotaxis after high-salt/food(+) and low-salt/food(–) conditioning.

**Fig 4:**
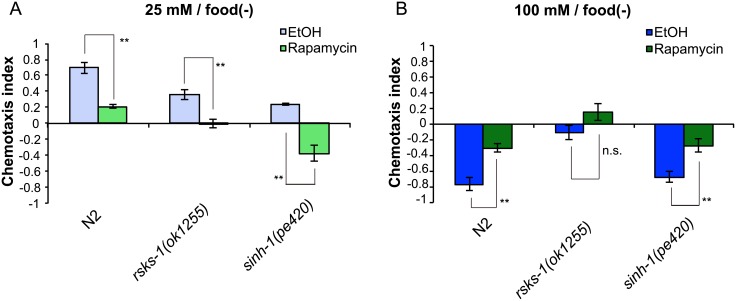
Effects of rapamycin on taste associative learning of CeTOR signaling mutants. (A and B) Salt chemotaxis of rapamycin-treated worms after starvation conditioning on low (A) or high (B) salt-containing agar plates; 100 μM rapamycin was used. Error bars, s.e.m.; *p < 0.05, **p < 0.01, n.s. = not significant (EtOH vs. rapamycin, Student’s *t* test, N ≥ 6).

To assess the mode of action of rapamycin on taste associative learning, we treated *rsks-1* and *sinh-1* mutants with rapamycin. Rapamycin administration enhanced the *sinh-1* phenotype after low-salt/food(–) conditioning (Fig 4A and 4B), which suggests that rapamycin inhibits migration to high salt levels through an unknown target other than TORC2. On the other hand, Rapamycin treatment did not cause statistically significant enhancement of the *rsks-1* phenotype after high-salt/food(–) conditioning (Fig 4A and 4B). These data are consistent with the hypothesis that RSKS-1 promotes migration to low salt levels after high-salt/food(-) conditioning, which is inhibited by rapamycin.

Because mutants of TORC2 components showed decreased migration to high salt concentrations, we suspected that these mutants have defects in salt sensation. We monitored the response of the ASER sensory neuron to salt stimuli by using the calcium indicator Yellow Cameleon. Because ASER responds to a stepwise decrease in NaCl concentrations [25], we examined ASER responses to a NaCl down-step from 50 to 25 mM in wild-type and *sinh-1* mutant worms after low-salt/food(–) conditioning, following which *sinh-1* mutants showed a defect in salt chemotaxis. We observed no statistically significant difference between wild-type and *sinh-1* mutant worms in ASER responses (Fig 3D and 3E), which suggests that animals lacking functional TORC2 can sense salt normally by ASER and that TORC2 might regulate sensory processing in ASER after salt sensation, or in the downstream neural circuit.

Our aforementioned results demonstrated that disruption of CeTOR and its putative substrates affect taste associative learning. To determine whether CeTOR signaling also regulates other types of learning, we analyzed the phenotypes of mutants of the CeTOR pathways in another associative learning paradigm, food-odor associative learning, in which the naive attractive response to the odorant benzaldehyde switches to avoidance after odor exposure in the absence of food [26]. Our analysis revealed that food-odor associative learning was defective in mutants of two TORC2 components, *rict-1* and *sinh-1*, but not in a *rsks-1* mutant (Fig 3F). We also investigated the rapamycin effect on food-odor associative learning and found that rapamycin treatment did not affect this type of learning (S5 Fig). The fact that mutants of TORC2 components showed reduced benzaldehyde avoidance after benzaldehyde exposure with starvation raises the possibility that CeTORC2 plays a general role in multiple types of behavioral plasticity. The *sinh-1* mutant showed diminished migration toward benzaldehyde even under naive conditions, which further suggests that TORC2 signaling might be involved in the sensation and/or processing of odor information. By contrast, the phenotype of the *rict-1(ft7)* mutant under naive conditions was not statistically different from that of wild-type worms, suggesting that *rict-1(ft7)*, but not *sinh-1(pe420)*, spares the TORC2 function required for odor chemotaxis.

### CeTORC2 functions in parallel with *akt-1* in taste associative learning

**Fig 5:**
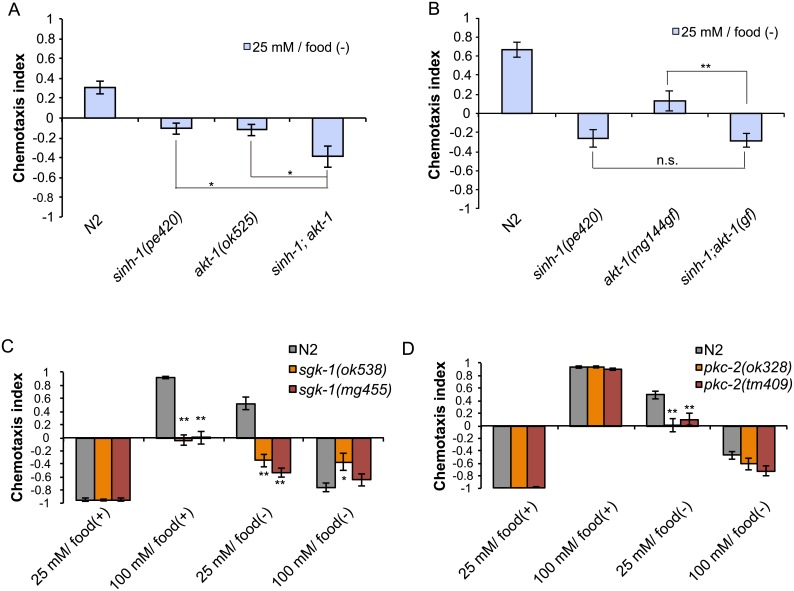
Putative TORC2 substrates *akt-1*, *pkc-2*, and *sgk-1* are involved in taste associative learning. (A) *sinh-1* and *akt-1* mutations have additive effects on salt chemotaxis after low-salt/food(–) conditioning. (B) *akt-1(mg144*gf) does not suppress the chemotaxis defect of *sinh-1(pe420)* after low-salt/food(–) conditioning. (C and D) Salt chemotaxis of the mutants of the putative TORC2 substrates *sgk-1* (C) and *pkc-2* (D). *sgk-1* mutants showed abnormal migration to low salt levels after 25 mM salt/food(–) and 100 mM salt/food(+) conditioning (C), whereas *pkc-2* mutants showed migration to low salt levels only after 25 mM salt/food(–) conditioning (D). Error bars, s.e.m.; *p < 0.05, **p < 0.01 (wild-type vs. each mutant, one-way ANOVA with Dunnett’s post hoc test, N ≥ 6).

TORC2 directly phosphorylates several members of the AGC kinase family, such as Akt1, SGK1, and PKC-α (Fig 3A) [4–6]. We have previously reported that *akt-1* mutants show defects in taste associative learning [11,12,17], and thus we hypothesized AKT-1 may be a CeTORC2 substrate in taste associative learning. We have reported that *akt-1(ok525*) mutants fail to avoid salt concentrations that were associated with starvation [11]. We hypothesized that if AKT-1 acts independently of CeTORC2, the CeTORC2 mutation would further enhance the learning defect of the *akt-1* mutant. Our analysis revealed that *sinh-1(pe420);akt-1(ok525)* double mutants migrated to lower salt levels than did *sinh-1(pe420)* or *akt-1(ok525)* single mutants after low-salt/food(-) conditioning (Fig 5A), which suggests that these mutations exert additive effects. We also tested the interaction between a gain-of-function mutation of *akt-1*, *akt-1(mg144*gf)[27], and *sinh-1(pe420)*, and found that the *akt-1(mg144*gf) mutation did not suppress the *sinh-1* phenotype (Fig 5B). These results argue against the possibility that AKT-1 functions as a CeTORC2 substrate in taste associative learning.

### Sgk and cPKC mutants show defects in taste associative learning

SGK1 was reported to be a substrate of TORC2 and to control the activities of ion channels and transporters by phosphorylating them [28]. Moreover, the SGK1 homolog in *C*. *elegans*, *sgk-1*, was reported to function downstream of TORC2 and play a role in controlling fat storage and aging [9,10,29]. Therefore, we examined the involvement of this candidate CeTORC2 substrate in taste associative learning. As observed in the case of TORC2 mutants, two *sgk-1* mutants migrated to lower salt concentrations than wild-type worms did after low-salt/food(–) and high-salt/food(+) conditioning (Fig 5C).

*pkc-2*, the only gene encoding conventional PKC (cPKC) in the *C*. *elegan*s genome, has been reported to function downstream of CeTORC2 [30]. We found that two *pkc-2* mutants also migrated to lower salt levels than did wild-type worms, but the phenotype of these mutants after low-salt/food(–) conditioning was weaker than those of other CeTORC2-pathway mutants (Fig 5D). Unlike most of the TORC2 mutants tested in this study, the *pkc-2* mutants showed no chemotaxis defect after high-salt/food(+) conditioning. Thus, PKC-2 appears to be involved in taste associative learning, but its contribution is less than that of other TORC2-pathway mutants.

We also tested whether these putative TORC2 substrates are involved in food-odor associative learning. Similar to the *rict-1(ft7)* mutant, the *sgk-1(ok538)* mutant showed weaker attraction to benzaldehyde under naive conditions and weaker avoidance after a 1-h exposure to benzaldehyde than did wild-type worms (Fig 3F). By contrast, both naive and conditioned *pkc-2(ok328)* mutants showed no marked defect in migration to benzaldehyde (Fig 3F). Thus, in contrast to its regulation of taste associative learning, *pkc-2* does not appear to contribute to associative learning between odor and starvation.

### *sgk-1* and possibly *pkc-2* function downstream of CeTORC2 to control taste associative learning

**Fig 6:**
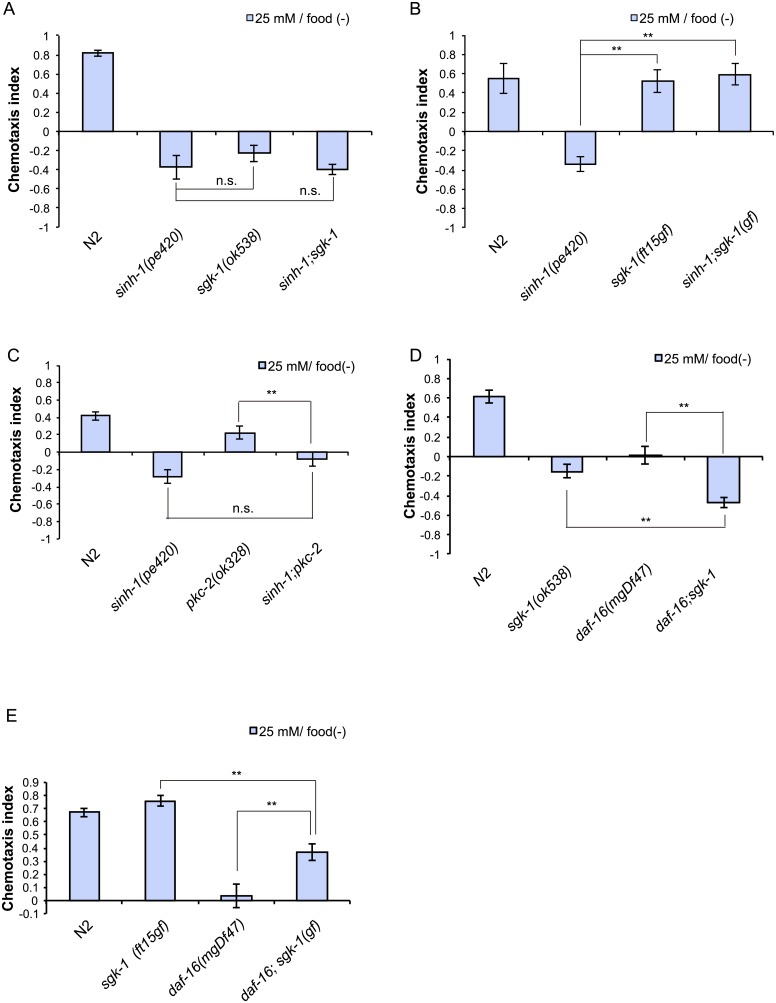
TORC2 and PKC-2 function in the same pathway and SGK-1 acts downstream of TORC2. (A) *sinh-1(pe420)* and *sgk-1(ok538)* double mutants do not show an additive effect on chemotaxis after low-salt/food(–) conditioning when compared to single mutants. (B) *sgk-1(ft15*gf) suppresses the *sinh-1(pe420)* chemotaxis defect after low-salt/food(–) conditioning. (C) *sinh-1(pe420)* and *pkc-2(ok328)* mutations do not produce an additive effect on salt chemotaxis after low-salt/food(–) conditioning. (D) *daf-16(mgDf47)* and *sgk-1(ok538)* have an additive effect on chemotaxis after low-salt/food(–) conditioning. (E) *sgk-1(ft15*gf) suppresses the chemotaxis defect of *daf-16(mgDf47)* after low-salt/food(–) conditioning. Error bars, s.e.m.; **p < 0.01, n.s. = not significant (one-way ANOVA with Dunnett’s post hoc test, N ≥ 6).

To test whether *sgk-1* and *pkc-2* act downstream of CeTORC2, we conducted genetic-interaction analyses. The deletion mutation allele *sgk-1(ok538)* did not enhance the chemotaxis defect of *sinh-1(pe420)* (Fig 6A), which suggests that *sinh-1* and *sgk-1* function in the same pathway to control taste associative learning. We next examined the TORC2-*sgk-1* interaction by using a putative gain of function allele of *sgk-1*, *sgk-1(ft15*gf), which was isolated through a genetic screen for suppressor mutations that block the high-fat-storage phenotype of a *rict-1* loss of function mutant [9,31]. The *sgk-1(ft15*gf) mutant showed largely normal salt chemotaxis (S6 Fig), and the mutation drastically suppressed the abnormal chemotaxis of the *sinh-1(pe420)* mutants (Fig 6B). These results indicate that *sgk-1* regulates taste associative learning downstream of CeTORC2. The *pkc-2(ok328)* mutation did not further enhance the abnormal migration to low salt concentrations of the *sinh-1(pe420)* mutant (Fig 6C), which is consistent with the notion that *pkc-2* functions in the same pathway as *sinh-1*.

We also investigated the interaction between *sgk-1* and *pkc-2*. The *pkc-2(ok328) sgk-1(ok538)* double mutant migrated to lower salt levels than the *sgk-1* or *pkc-2* single mutants did after low-salt/food(−) conditioning (S7 Fig), which suggests that these two genes function in parallel. Collectively, the results of the genetic-interaction analyses support the idea that *sgk-1* and *pkc-2* act in parallel downstream of TORC2. However, we cannot exclude the possibility that *pkc-2* functions independently of TORC2 signaling.

### *sgk-1* functions in parallel with *daf-16* in taste associative learning

Mammalian SGK has been reported to directly phosphorylate a forkhead box O (FOXO) transcription factor and inhibit its activation [32]. In *C*. *elegans*, SGK-1 positively regulates DAF-16, the sole FOXO homolog [33], and thereby controls longevity [31,34]. Therefore, we investigated the genetic interaction between *daf-16* and *sgk-1*. After low-salt/food(–) conditioning, the double mutant *daf-16(mgDf47);sgk-1(ok538)* showed a more severe phenotype than the single mutants did (Fig 6D), which indicates that DAF-16 and SGK-1 exert additive effects. Conversely, the gain of function mutation *sgk-1(ft15*gf) partially suppressed the phenotype of the *daf-16* null mutant *daf-16(mgDf47)* (Fig 6E). If *daf-16* functioned as the sole downstream effector of *sgk-1*, *sgk-1*(gf) would not be expected to suppress the chemotaxis defect of the *daf-16* mutant. Collectively, these results suggest that *daf-16* and *sgk-1* act in parallel in taste associative learning.

### *pkc-2* functions in neurons and *sgk-1* functions in the intestine to regulate taste associative learning

Our results thus far demonstrated that the mutants of the CeTORC2 components and the *sgk-1* and *pkc-2* mutants showed abnormal taste associative learning, particularly after starvation conditioning. To identify the sites of action of these genes, we performed tissue-specific rescue experiments.

**Fig 7:**
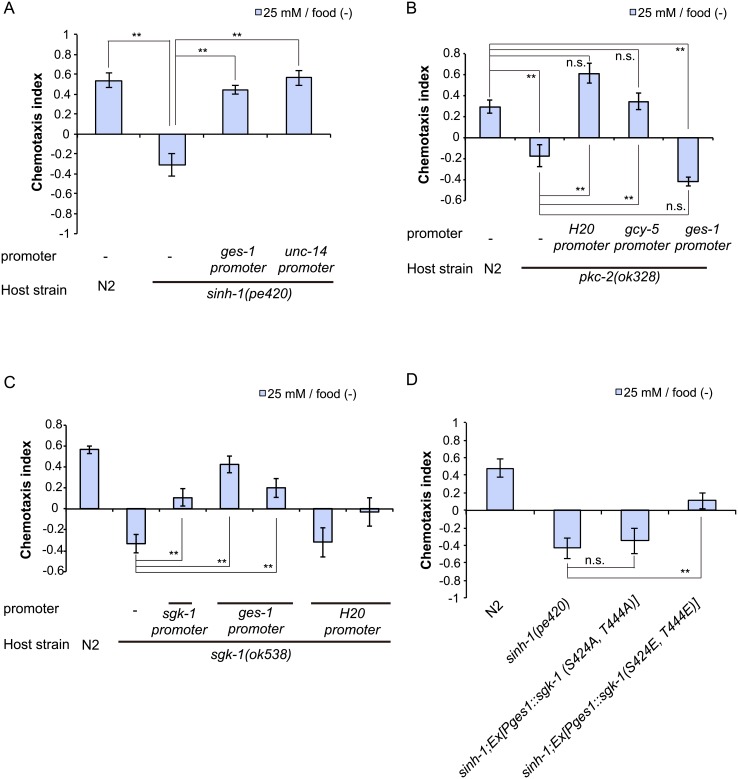
TORC2/PKC-2 pathway functions in neurons, whereas TORC2/SGK-1 pathway functions in the intestine. (A) Neuronal (*unc-14* promoter) and intestinal (*ges-1* promoter) expression of *sinh-1* cDNA rescued the chemotaxis defects of the *sinh-1(pe420)* mutant after low-salt/food(–) conditioning. (B) Pan-neuronal expression (*H20* promoter) and ASER-specific expression (*gcy-5* promoter), but not intestine-specific expression (*ges-1* promoter), of the *pkc-2* cDNA rescued the chemotaxis abnormality of the *pkc-2(ok328)* mutant after low-salt/food(–) conditioning. (C) Intestine-specific expression (*sgk-1B* promoter or *ges-1* promoter), but not pan-neuronal expression (*H20* promoter), of *sgk-1* isoform B was sufficient for rescuing the chemotaxis defect of the *sgk-1(ok538)* mutant after low-salt/food(–) conditioning. (D) Intestine-specific expression of the active form of *sgk-1* suppressed the chemotaxis defect of the *sinh-1(pe420)* mutant after low-salt/food(–) conditioning. Error bars, s.e.m.; *p < 0.05, **p < 0.01, n.s. = not significant (Dunnett’s test, N ≥ 9).

First, we investigated the site of function of *sinh-1*, the CeTORC2 component. Interestingly, intestinal expression of *sinh-1* cDNA was sufficient for rescuing the *sinh-1* chemotaxis defect (Fig 7A), which suggested that intestinal CeTORC2 functions contribute to taste associative learning. Furthermore, neuronal expression of the cDNA under a pan-neuronal promoter, the *unc-14* promoter, also rescued the defect of the *sinh-1* mutant (Fig 7A). Therefore, *sinh-1* might act not only in the intestine, but also in neurons. However, we observed that in addition to driving neuronal expression, the *unc-14* promoter drove weak expression in the intestine. Therefore, we cannot exclude the possibility that this weak expression in the intestine was sufficient for the rescue of the *sinh-1* phenotype observed with the use of the *unc-14* promoter.

We next investigated the sites of action of *pkc-2* and *sgk-1*, the putative downstream components of TORC2. Previous works have shown that *pkc-2* is expressed in multiple neurons, muscle cells, the gonads, and the intestine [35,36], and we found that expression of the *pkc-2* cDNA in neurons or the ASER neuron, but not in the intestine, rescued the chemotaxis defect of *pkc-2* mutants (Fig 7B); this result suggests that *pkc-2* functions in ASER to regulate taste associative learning.

The translational reporter construct of *sgk-1* was reported to be expressed mainly in several neurons and the intestine [37]. Therefore, we expressed the *sgk-1* cDNA in neurons or the intestine of *sgk-1(ok538)* mutants. Intestine-specific *sgk-1* expression under the *ges-1* promoter rescued the chemotaxis defect of *sgk-1(ok538)* (Fig 7C). Moreover, *sgk-1* expression driven by the pan-neuronal *H20* promoter exerted a weak, but non-significant rescue trend on the *sgk-1* defect. These results suggested the importance of intestinal CeTORC2/SGK-1 signaling in associative learning. We performed additional rescue experiments by using the *sgk-1A* and *sgk-1B* promoters, both of which drove expression only in the intestine (S8A–S8D Fig). The differences in the expression patterns observed here and in a previous study [37] that reported neuronal expression of *sgk-1* might be explained by the use of not only the upstream region of *sgk-1*, but also exonic and intronic regions in the previous study. The *sgk-1* expression driven by these promoters rescued the *sgk-1* defects (Fig 7C, S8E Fig), which further supports the notion that intestinal SGK-1 functions in learning.

Lastly, to ascertain whether SGK-1 phosphorylation is involved in learning, we constructed mutant *sgk-1* cDNAs that included unphosphorylatable and phosphomimetic substitutions. mTORC2 phosphorylates the hydrophobic motif (HM) and the turn motif (TM) of AGC kinases such as Akt1, PKC, and SGK1 [5,38]. We introduced substitution mutations at S424 in the TM and T444 in the HM of SGK-1, the residues equivalent to S379 and S424 in mouse SGK1, respectively [9]. Whereas expression of the cDNA encoding the unphosphorylatable *sgk-1* (S424A, T444A) in the *sinh-1* mutant produced no effect (Fig 7D), intestine-specific expression of the phosphomimetic *sgk-1* cDNA (S424E, T444E) partially suppressed the abnormal chemotaxis of *sinh-1* mutants (Fig 7D). These results are consistent with the possibility that direct phosphorylation of SGK-1 by CeTORC2 in the intestine is critical for taste associative learning.

## Discussion

Since the discovery of *C*. *elegans* Raptor *daf-15* [39] and *C*. *elegans* Rictor *rict-1* [9,10], the functions of these molecules in development, metabolism, and longevity have been extensively studied [9,10,14,31,40]. By contrast, the roles of CeTOR signaling in neural functions have remained poorly investigated. In this study, we characterized for the first time the functions of CeTOR signaling in associative learning. Our results indicate that the functions of both CeTORC1 and CeTORC2 in neuronal or non-neuronal cells contribute to behavioral changes induced by salt and food availability. Although mammalian TOR signaling is recognized to be involved in neural functions such as long-term memory formation and neuronal excitation [7,8,41,42], the mechanistic details of mTOR control of neuronal activity remain unclear. Given that TOR signaling is highly conserved across species, elucidation of the role of CeTOR signaling in the regulation of neuronal functions will contribute toward the understanding of TOR signaling in other species.

The results of our genetic analyses suggest that CeTORC1 and CeTORC2 control learning-dependent behaviors in *C*. *elegans*. We evaluated the behaviors of the CeTOR signaling mutants using chemotaxis assays after four kinds of salt conditioning: attraction to high and low salt concentrations associated with food and avoidance of high and low salt concentrations associated with starvation (S1 Table). We demonstrated that neuronal RSKS-1, the putative CeTORC1 substrate, contributes to avoidance of high salt concentrations after high-salt/food(–) conditioning (Fig 2B, S1 Table). Previous studies have reported that mTORC1/S6K signaling plays critical roles in protein synthesis in neurons, where it contributes to synaptic plasticity and is essential for neuronal function [43–45]. Thus, in *C*. *elegans*, CeTORC1 might also control synaptic functions by regulating protein synthesis. On the other hand, mutants of CeTORC2-defining components RICT-1 and SINH-1, and its putative substrate SGK-1, showed decreased migration to high salt levels and/or increased migration to low salt levels after high-salt/food(+) and low-salt/food(–) conditioning (Fig 3C–3E, S1 Table), indicating that CeTORC2/SGK-1 signaling contributes to migration towards high salt concentrations. These defects were rescued by the intestinal expression of each of the responsible genes (Fig 7A, 7C and 7D). Our results obtained using the *sgk-1(gf)* mutant and the intestinal expression of phosphomimetic SGK-1 suggested that SGK-1 functions downstream of TORC2 in taste associative learning. Thus, CeTORC1 and CeTORC2 signaling pathways contribute to generating appropriate behavioral responses after learning by acting in different tissues, the nervous system and the intestine, respectively(S9 Fig).

Besides the above-mentioned mechanism, TORC1 and TORC2 signaling components appear to have additional functions in taste associative learning. Among the TORC2 signaling mutants tested in this study, only the *sgk-1(ok538)* mutant showed a defect in avoidance of high salt after high-salt/food(−) conditioning. Because SGK-1 is also regulated by insulin-like signaling, [37] and insulin-like pathway mutants showed strong defects in avoidance of high salt after high-salt/food(−) conditioning [11], SGK-1 might also promote migration to low salt after starvation conditioning through insulin-like signaling. Furthermore, there might also be some interactions between TORC1 and TORC2 pathways in neurons and the intestine. PKC-2 controls low salt avoidance possibly in the CeTORC2 pathway after low-salt/food(−) conditioning in neurons (Fig 7B, S1 Table), where PKC-2 might interact with the CeTORC1 signaling pathway. Although we have not determined the site of action of ATG-13 and its possible interaction with CeTORC1 in taste associative learning, the similar learning phenotypes of the *atg-13* and CeTORC2 mutants (S1 Table) imply that ATG-13 and CeTORC2/SGK-1 pathways might control the same process in the intestine. These possibilities should be further investigated in future studies.

The analysis performed using the *sinh-1;pkc-2* double mutant suggested that TORC2 and PKC-2 act in the same genetic pathway. In food-odor associative learning, the *sgk-1* mutant showed a phenotype similar to those of TORC2-component mutants, whereas the *pkc-2* mutant showed no notable defect (Fig 3F). Recently, PKC-α signaling was shown to control long-term memory formation through neuronal actin organization in mice and *Drosophila melanogaster* [7]. Intriguingly, the neuronal function of ADD-1, a *C*. *elegans* homolog of α-adducin, which promotes assembly of the spectrin-actin cytoskeleton, is required for several forms of associative learning [46]. Thus, in *C*. *elegans*, the neuronal PKC-2 pathway might function by regulating the actin cytoskeleton as in mouse and *Drosophila* neurons. It will be of interest to investigate how SGK-1 and PKC-2 regulate intestinal and neuronal functions, respectively, and clarify the roles of SGK-1 and PKC-2, including their target molecules, in TORC2 signaling in associative learning.

How could TORC2/SGK-1 signaling acting in the intestine control the neuronal functions that underlie chemotaxis behavior? Given that the intestine is a major organ for nutrient uptake and the TOR pathway is recognized as a nutrition sensor, it’s possible that TORC2/SGK-1 signaling functions as a mediator of nutrient signals. If this is the case, loss of *sgk-1* and TORC2 components would result in defective chemotaxis after fed conditioning. Interestingly, mutants of *sgk-1* and TORC2 components showed defects in migration to high, but not low, salt levels after fed conditioning. Furthermore, these mutants also showed defective migration to high salt levels after starvation conditioning. These observations imply that TORC2/SGK-1 signaling transmits salt-related information to promote migration to high salt levels after learning. One possibility is that intestinal TORC2/SGK-1 signaling acts in salt sensation. Previous studies have shown that non-neural organs, including the intestine, could also function as sensors for environmental conditions such as temperature [34,47]. In taste associative learning, the intestine might act as a chemical sensor of salt concentration and transmit information onto neurons through, for example, secretion of a hormone-like substance, to promote migration to high salt concentrations. Another possibility is that the *sgk-1* phenotype is caused by changes in lipid metabolism. The results of previous studies and those obtained here using Oil-Red-O staining suggest that mutants of *rict-1*, *sinh-1*, and *sgk-1* accumulate more lipid in the intestine than do wild-type animals [9,10] (S4 Fig). Moreover, we previously reported that mutants of a PTEN homolog, *daf-18*, in which PI3-kinase signaling is activated, and a novel protein kinase C-ε/η ortholog, *pkc-1*, in which the Gq/DAG/PKC pathway is inactivated, showed defective migration to high salt levels after starvation or fed conditioning similar to the *sgk-1* and the TORC2-component mutants [11,12]. Both of these signaling pathways use lipids, such as phosphatidylinositols and diacylglycerol, as second messengers [11–13]. Dysfunction of intestinal SGK-1 might affect lipid metabolism not only in the intestine but also in neurons, and lead to altered actions of the PI3-kinase pathway or the Gq/DAG/PKC pathway, both of which are required for normal salt chemotaxis.

Rapamycin, which is used as an immunosuppressive drug, has drawn considerable attention because of its ability to increase longevity and its usefulness in the treatment of various diseases [48,49]. In relation to neural functions, chronic oral administration of rapamycin has been shown to exert beneficial effects on learning and memory [50–53]. However, certain studies have reported that direct rapamycin administration into the central nervous system impairs long-term potentiation in the hippocampus or learning ability [41,43,44,50,54–56]. In this study, we investigated the rapamycin effect only during conditioning and chemotaxis tests. This is considered an acute rapamycin effect rather than a chronic effect. This result agrees with the findings of previous studies showing that acute local administration of rapamycin impairs learning and memory formation [41,43,44,50,54–56]. Although rapamycin was previously reported to be capable of crossing the blood-brain barrier [57], the general whole-body effects of rapamycin and the local effect of rapamycin on the brain should be distinguished because the concentration and localization of rapamycin are considered to differ following peroral versus regional administration. Further investigation is required for a complete understanding of the beneficial and deleterious effects of rapamycin as a therapeutic compound.

The result obtained with rapamycin treatment of the *sinh-1* mutants implies that an unknown rapamycin target other than TORC2 is involved in the learning paradigm studied here. Because the rapamycin concentration used here was high, unexpected side effects which have not been reported in other animals may have been observed. Exploiting the forward genetic screening possible in *C*. *elegans* might facilitate the identification of previously unrecognized rapamycin targets.

In this study, we characterized for the first time the functions of CeTORC2 signaling in learning. The simple nervous system of *C*. *elegans* and the viability of CeTORC2-component mutants will help us elucidate the functions of TORC2 in the nervous system. Therefore, future analysis of CeTORC2 using our experimental model may help clarify previously unknown conserved functions of TORC2 in learning.

## Materials and methods

### Strains and culture

Animals were grown at 20°C on *Escherichia coli* strain NA22 as a food source under standard conditions [58]. N2 Bristol was used as the wild-type strain. The *C*. *elegans* mutant and transgenic strains used in this study were as follows: *Ex[P*_*gcy-5*_::*YC2*.*60;P*_*lin-44*_::*mCherry]*, *Ex[P*_*H20*_::*RSKS-1(F5A-T404E -R3A);P*_*myo3*_::*venus]*, *Ex[P*_*sgk-1A*_::*venus]*, *Ex[P*_*sgk-1B*_::*venus]*, *daf-16(mgDf47)I*, *daf-16(mgDf47)I;sgk-1(ok538)X*, *daf-16(mgDf47)I;sgk-1(ft15)X*, *rict-1(ft7)II*, *rict-1(tm4017)II*, *rict-1(mg360)II*, *sinh-1(pe420)II*, *sinh-1(pe420)II;akt-1(ok525)V*, *sinh-1(pe420)II;akt-1(mg144)V*, *sinh-1(pe420)II;pkc-2(ok328)X*, *sinh-1(pe420)II;sgk-1(ok538)X*, *sinh-1(pe420)II;sgk-1(ft15)X*, *sinh-1(mg360);Ex[P*_*ges-1*_::*sgk-1(S424A*,*T444A);P*_*myo-3*:_:*venus]*, *sinh-1(mg360);Ex[P*_*ges-1*_::*sgk-1(S424E*,*T444E);P*_*myo-3*_::*venus]*, *sinh-1(mg360);Ex[P*_*H20*_::*pkc-2CAT;P*_*myo-3*_::*venus]*, *sinh-1(pe420)II;Ex[P*_*ges-1*_::*sinh-1;P*_*myo-3*_::*venus]*, *sinh-1(pe420)II;Ex[P*_*unc-14*_::*sinh-1;P*_*myo-3*_::*venus]*, *sinh-1(pe420)II;Ex[P*_*gcy-5*_::*YC2*.*60;P*_*lin-44*_::*mCherry]*, *rsks-1(ok1255)III*, *rsks-1(ok1255)III;Ex[P*_*H20*_::*rsks-1;P*_*myo-3*_::*venus]*, *rsks-1(ok1255)III;Ex[P*_*gcy-5*_::*rsks-1;P*_*myo-3*_::*venus]*, *rsks-1(ok1255)III;Ex[P*_*ges-1*_::*rsks-1;P*_*myo-3*_::*venus]atg-13(bp414)III*, *atg-13(bp417)IV;bpIs267[PY37A1B*.*5*::*sqst-1*::*gfp+unc-76]V*, *akt-1(ok525)V*, *akt-1(mg144)V*, *pkc-2(ok328)X*, *pkc-2(tm409)X*, *pkc-2(ok328)X;Ex[P*_*H20*_::*pkc-2;P*_*myo3*_::*venus]*, *pkc-2(ok328)X;Ex[P*_*gcy-5*_::*pkc-2;P*_*myo-3*_::*venus]*, *pkc-2(ok328)X;Ex[P*_*ges-1*_::*pkc-2cDNA;P*_*myo-3*_::*venus]*, *pkc-2(ok328) sgk-1(ok538)X*, *sgk-1(mg455)X*, *sgk-1(ft15)X*, *sgk-1(ok538)X*, *sgk-1(ok538)X;Ex[P*_*sgk-1A*_::*sgk-1A;P*_*myo-3*_::*venus;]*, *sgk-1(ok538)X;Ex[P*_*sgk-1B*_::*sgk-1B;P*_*myo-3*_::*venus;]*, *sgk-1(ok538)X;Ex[P*_*ges-1*_::*sgk-1B;P*_*myo-3*_::*venus]*, *sgk-1(ok538)X;Ex[P*_*H20*_::*sgk-1B;P*_*myo-3*_::*venus]*

### Statistical analyses

All data, except for the lifespan assay, were analyzed by Student’s *t* test or one-way ANOVA with Dunnett’s post-hoc test using Graph-pad Prism 5.0 statistical software (Graph-pad Software, San Diego, CA).

### Behavioral assays

The salt-chemotaxis learning assays were performed as described previously [11]. Briefly, adult animals grown on standard nematode growth medium (NGM) were transferred for conditioning to NGM plates that contained NaCl at low (25 mM) or high (100 mM) concentration together with or without food. After 5–6 h, animals were washed out from the plates with chemotaxis buffer (50 mM NaCl, 25 mM potassium phosphate (pH 6.0), 1 mM CaCl_2_, 1 mM MgSO_4_), placed at the center of test plates featuring a salt gradient, and allowed to run for 40 min. The chemotaxis index was calculated as (A—B)/(N—C), where A is the number of animals within 2 cm of the highest point of the salt gradient, B is the number of animals within 2 cm of the lowest point of the salt gradient, N is the total number of animals on a test plate, and C is the number of animals that remained in the central region (S1B Fig).

The food-odor associative learning assays were performed using benzaldehyde, as described with modifications [59]. To test the odor chemotaxis of naive animals, well-fed worms were washed thrice with chemotaxis buffer (5 mM potassium phosphate (pH 6.0), 1 mM CaCl_2_, 1 mM MgSO_4_), placed at the center of test plates featuring a benzaldehyde gradient, and allowed to run for 30 min. To prepare the test plates with the benzaldehyde gradient, 1 μL of benzaldehyde (1:100 dilution) was spotted on each of two points at one end of the plates, 1 μL of ethanol was spotted on each of two points at the other end of the plates, and 1 μL of 0.5 M NaN_3_ was spotted on two points at both ends of the plates to anesthetize worms. For odor adaptation, animals were soaked in 500 μL of benzaldehyde (1:10^5^ dilution) for 1 h, and after washing thrice with chemotaxis buffer, were placed at the center of the test plates and allowed to run for 30 min. Chemotaxis index for the olfactory-plasticity assays was calculated as (A—B)/(A + B), where A is the number of animals on the odorant-spotted side of the plate and B is the number of animals on the opposite side (S1C Fig).

In each behavioral experiment, we simultaneously performed behavioral assays with indicated conditions and worm strains and repeated the assays at least four times.

### Plasmid constructions and germ-line transformation

All plasmid constructs were generated using the GATEWAY system (Invitrogen). To construct entry vectors harboring the promoter sequences of *sgk-1*, 2.0 kb upstream of the first exon of *sgk-1* isoform A and 2.5 kb upstream of the first exon of *sgk-1* isoform B were amplified from *C*. *elegans* genomic DNA, and then inserted into an entry vector, pENTR1A. The *rsks-1*, *pkc-2*, *sgk-1*, and *sinh-1* cDNAs were cloned from *C*. *elegans* total cDNA, and inserted into a destination vector, pPD-DEST, to generate pDEST-*rsks-1*, pDEST-*pkc-2*, pDEST-*sgk-1*, and pDEST-*sinh-1*, respectively. To construct pDEST-*rict-1*, *rict-1* cDNA (a kind gift from S. Takagi) was inserted into the destination vector pPD-DEST.

Germ-line transformation was performed using standard microinjection techniques, as described previously [60]. In rescue experiments, pG-*myo-3p*::*Venus* was used as a co-injection marker. The injection mixtures contained 10 ng/μL of each plasmid DNA.

### Generation of *sinh-1(pe420)* mutant strain

The *pe420* allele of *sinh-1* was generated using the CRISPR-Cas9 system, as described previously [61]. The expression constructs of sgRNAs containing the target sequences 5'-GACAGGAAATGTCGTGCCGG-3', 5'-GACATTTCCTGTCTGGATTC-3', and 5'-GGCTTCTCCTGAATCCAGAC-3' were prepared using a PCR-fusion technique and injected at 10 ng/μL each together with 50 ng/μL pDD162-P*eft-3*::*Cas9*. After injection, F1 animals were screened by means of single-worm PCR performed using the primers 5'-ACGTAATTCGACATGAGCTTC-3' and 5'-ATATCATCAAATCCCGCATCC-3'.

### Rapamycin administration

Rapamycin (LC laboratories) was dissolved in 10% Tween-20/90% ethanol, and 20 h before assays, rapamycin was added to conditioning plates or test plates at a final concentration of 100 μM. Control plates were prepared to contain the solvents at the same concentration as that in the rapamycin-containing plates.

### Ca^2+^ imaging

We used animals expressing YC2.60 in the ASER neuron to visualize calcium ion dynamics in response to NaCl stimuli. Imaging experiments were performed as previously reported [11] with modification. In this study, animals were conditioned on normal NGM plates without glycerol.

### Body fat assessment

Oil-Red-O staining was conducted as described previously [62]; images of stained animals were acquired using a Zeiss Axiovert S100 microscope and a CCD camera (Keyence VB-7010). To quantify Oil-Red-O staining, we manually determined the region of interest, which included the most of the anterior body region extending from the posterior edge of the pharynx to the vulva (S4B Fig) in each animal, and then measured red, green, and blue (RGB) intensities by using Image J software. Body fat levels were calculated as ratios of red to green intensities after subtraction of intensities measured in background regions.

## Fluorescence microscopy

Well-fed adult worms were mounted on a 5% agar pad with 10 mM NaN_3_ and observed under a Leica confocal microscope (TCS-SP5).

## Supporting information

S1 Fig Salt chemotaxis of wild-type N2 worms. (A) Wild-type *C*. *elegans* migrated to the NaCl concentration at which they were previously cultivated with food (orange bars). By contrast, the worms avoided the salt level at which they previously experienced starvation (blue bars). High and low chemotaxis indices indicate that the worms migrated to high and low salt levels, respectively. (B) Schematic diagram of a salt-chemotaxis test plate. The chemotaxis index was calculated as (N[A]–N[B])/(N[all]–N[C]), where N[A] is the number of animals within a 2 radius cm of the highest point in the salt gradient, N[B] is the number of animals within a 2 cm radius of the lowest point in the salt gradient, N[all] is the total number of animals on the test plate, and N[C] is the number of animals that remained in the central region. (C) Schematic diagram of a benzaldehyde-chemotaxis test plate. The chemotaxis index was calculated as (N[A]–N[B])/(N[A] + N[B]), where N[A] is the number of animals on the odorant-spotted side of the plate and N[B] is the number of animals on the opposite side.
(TIF)

S2 Fig Taste associative learning of *rsks-1*, *sinh-1*, and *rict-1* mutants. A replicate experiment of those shown in Figs 2B and 3B. Taste associative learning of N2, *rsks-1*, *sinh-1*, and *rict-1* mutants were assayed simultaneously. Error bars, s.e.m.; **p < 0.01 (Dunnett’s test, N ≥ 9).
(TIF)

S3 Fig Genomic structure of TORC2 pathway-related genes. Genomic structure of *rict-1* (top), *pkc-2* (middle), and *sgk-1* (bottom). Black boxes indicate exons, red boxes indicate deletion mutations, and arrowheads indicate point mutations.
(TIF)

S4 Fig Body fat is increased in the *sinh-1* mutant. (A) Examples of Oil-Red-O-stained animals. Scale bars: 100 μm. (B) To quantify Oil-Red-O staining, we determined the R/G ratios of the region of interest, which included most of the anterior portion of the body: from the posterior edge of the pharynx to the vulva in each animal (indicated with a purple box). The details are listed in Materials and Methods. (C) Quantification of Oil-Red-O staining in wild-type, *rict-1(ft7)*, and *sinh-1(pe420)* animals. As previously reported based on experiments conducted using Nile-Red staining [9], our Oil-Red-O staining results demonstrated a body fat increase in *rict-1(ft7)* animals. Similar to the *rict-1(ft7)* animals, *sinh-1(pe420)* animals also showed the increased body fat phenotype, which is in agreement with previous reports that Sin1 is an essential component of TORC2 in several species [22–24]. Data were normalized against the averaged value determined for wild-type. Error bars, s.e.m.; **p < 0.01 (wild-type vs. each mutant, Dunnett’s test, N = 40).
(TIF)

S5 Fig Rapamycin treatment does not affect food-odor associative learning. Rapamycin administration didn’t affect food-odor associative learning. This is consistent with the phenotype of *rsks-1(ok1255)* mutants, which showed no defect in food-odor associative learning. The learning assay was performed using benzaldehyde as an odorant as described in Fig 4F. Error bars, s.e.m.; n.s. = not significant (Student’s *t* test, N ≥ 8).
(TIF)

S6 Fig The *sgk-1*(gf) mutant shows largely normal taste associative learning. Chemotaxis of the *sgk-1(gf)* mutant after salt conditioning is shown. Error bars, s.e.m.; *p < 0.05, n.s. = not significant (Student’s *t* test, N ≥ 6).
(TIF)

S7 Fig SGK-1 and PKC-2 function in parallel in taste associative learning. *sgk-1;pkc-2* double mutants showed enhanced migration to low salt levels as compared with each single mutant. Error bars, s.e.m.; **p < 0.01 (Dunnett’s test, N ≥ 9).
(TIF)

S8 Fig Both A and B isoforms of *sgk-1* are expressed in the intestine. (A) Venus expression driven by the *sgk-1* isoform A promoter (2.0 kb upstream of the first exon of *sgk-1* isoform A). The *sgk-1* isoform A promoter drives strong expression in the intestine, but not in neurons. Left, bright field; center, Venus; right, merged. Scale bar: 100 μm. (B) No signal was detected in the head ganglia. Scale bar: 30 μm. (C) Venus expression driven by the *sgk-1* isoform B promoter (2.5 kb upstream of the first exon of *sgk-1* isoform B). The *sgk-1* isoform B promoter drives expression in the intestine. Scale bar: 100 μm. (D) No fluorescence was detected in the head ganglia. Scale bar: 30 μm. (E) Intestine-specific expression (*sgk-1A* promoter) of *sgk-1* isoform A was sufficient for rescuing the chemotaxis defect of the *sgk-1(ok538)* mutant after low-salt/food(–) conditioning. Error bars, s.e.m.; **p < 0.01 (Dunnett’s test, N ≥ 9).
(TIF)

S9 Fig A model for TORC1 and TORC2 regulation in taste associative learning. Intestinal TORC2/SGK-1 signaling and neuronal RSKS-1 and PKC-2 contribute to taste associative learning: TORC2/SGK-1 promotes migration to high salt levels through SGK-1 phosphorylation in the intestine after fed and starvation conditioning.
(TIF)

S1 Table Summary of behavioral phenotypes of CeTOR signaling mutants. (TIF)
