# Supplementary figures and images for: The intestinal TORC2 signaling pathway contributes to associative learning in Caenorhabditis elegans
Source: PLoS One. 2017 May 25;12(5):e0177900. doi: 10.1371/journal.pone.0177900 (PMC5444632; doi:10.1371/journal.pone.0177900)

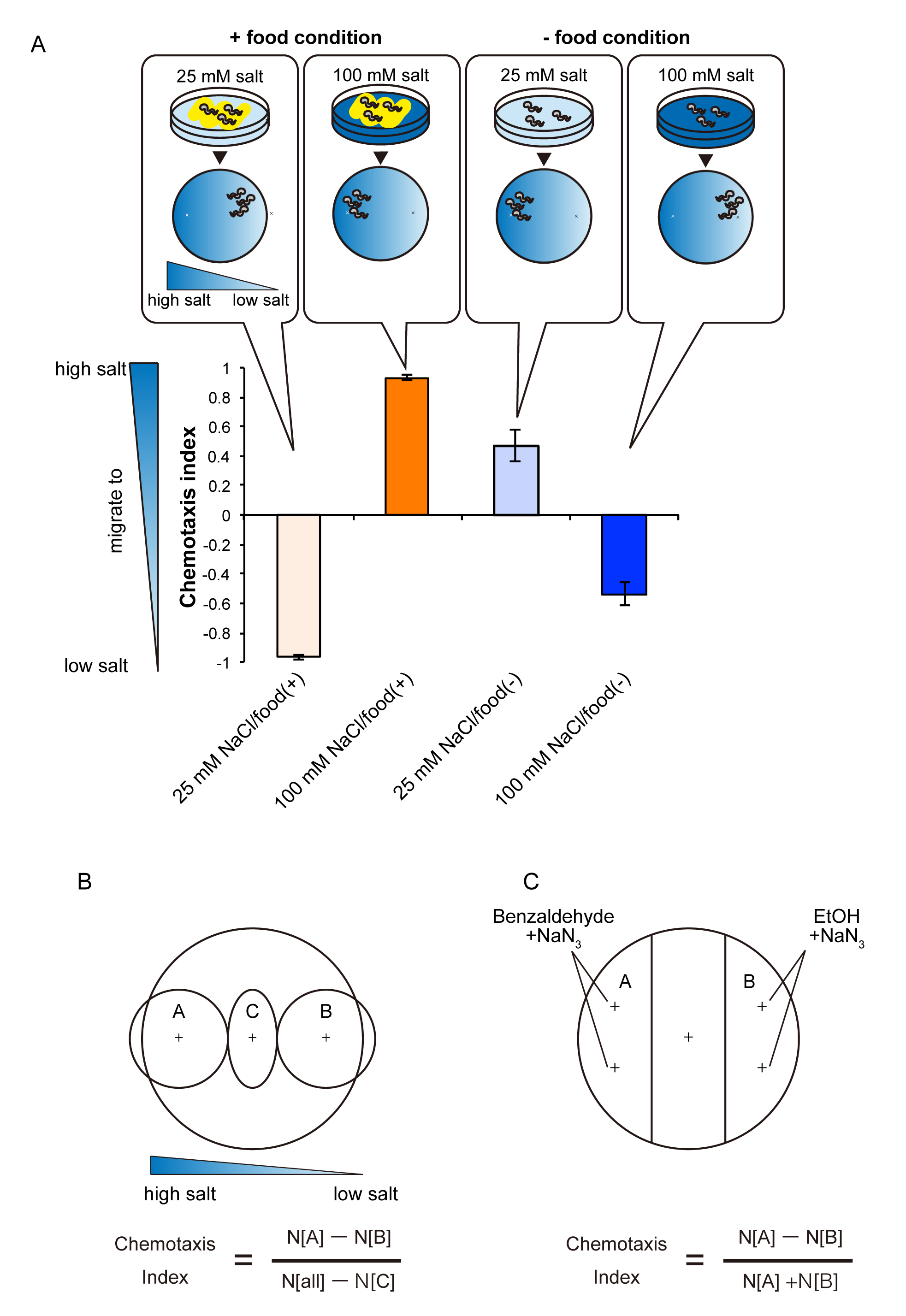

Supplement: S1 Fig — (A) Wild-type C. elegans migrated to the NaCl concentration at which they were previously cultivated with food (orange bars). By contrast, the worms avoided the salt level at which they previously experienced starvation (blue bars). High and low chemotaxis indices indicate that the worms migrated to high and low salt levels, respectively. (B) Schematic diagram of a salt-chemotaxis test plate. The chemotaxis index was calculated as (N[A]–N[B])/(N[all]–N[C]), where N[A] is the number of animals within a 2 radius cm of the highest point in the salt gradient, N[B] is the number of animals within a 2 cm radius of the lowest point in the salt gradient, N[all] is the total number of animals on the test plate, and N[C] is the number of animals that remained in the central region. (C) Schematic diagram of a benzaldehyde-chemotaxis test plate. The chemotaxis index was calculated as (N[A]–N[B])/(N[A] + N[B]), where N[A] is the number of animals on the odorant-spotted side of the plate and N[B] is the number of animals on the opposite side. (TIF) [file pone.0177900.s001.tif]

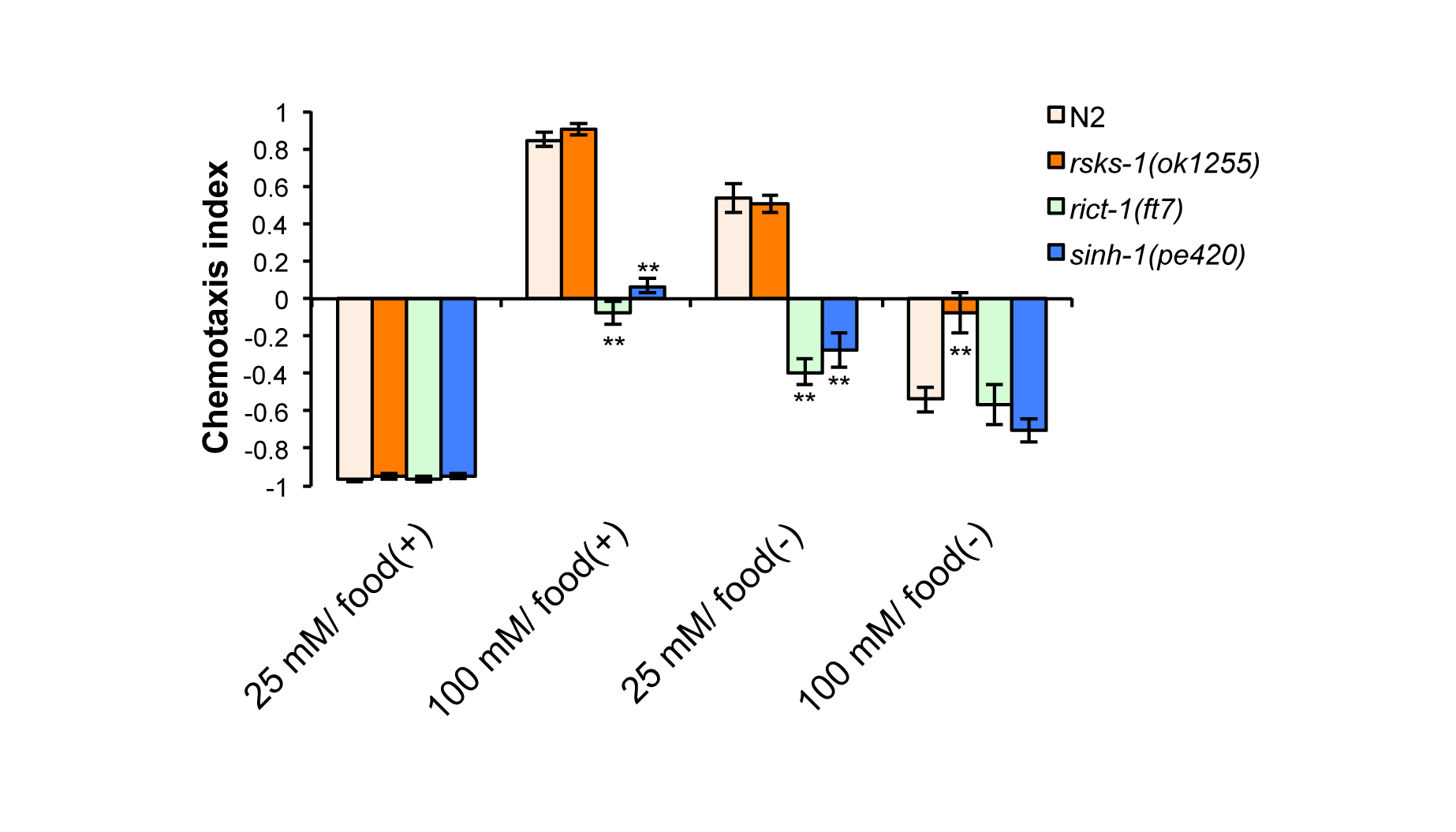

Supplement: S2 Fig — A replicate experiment of those shown in Figs 2B and 3B. Taste associative learning of N2, rsks-1, sinh-1, and rict-1 mutants were assayed simultaneously. Error bars, s.e.m.; **p < 0.01 (Dunnett’s test, N ≥ 9). (TIF) [file pone.0177900.s002.tif]

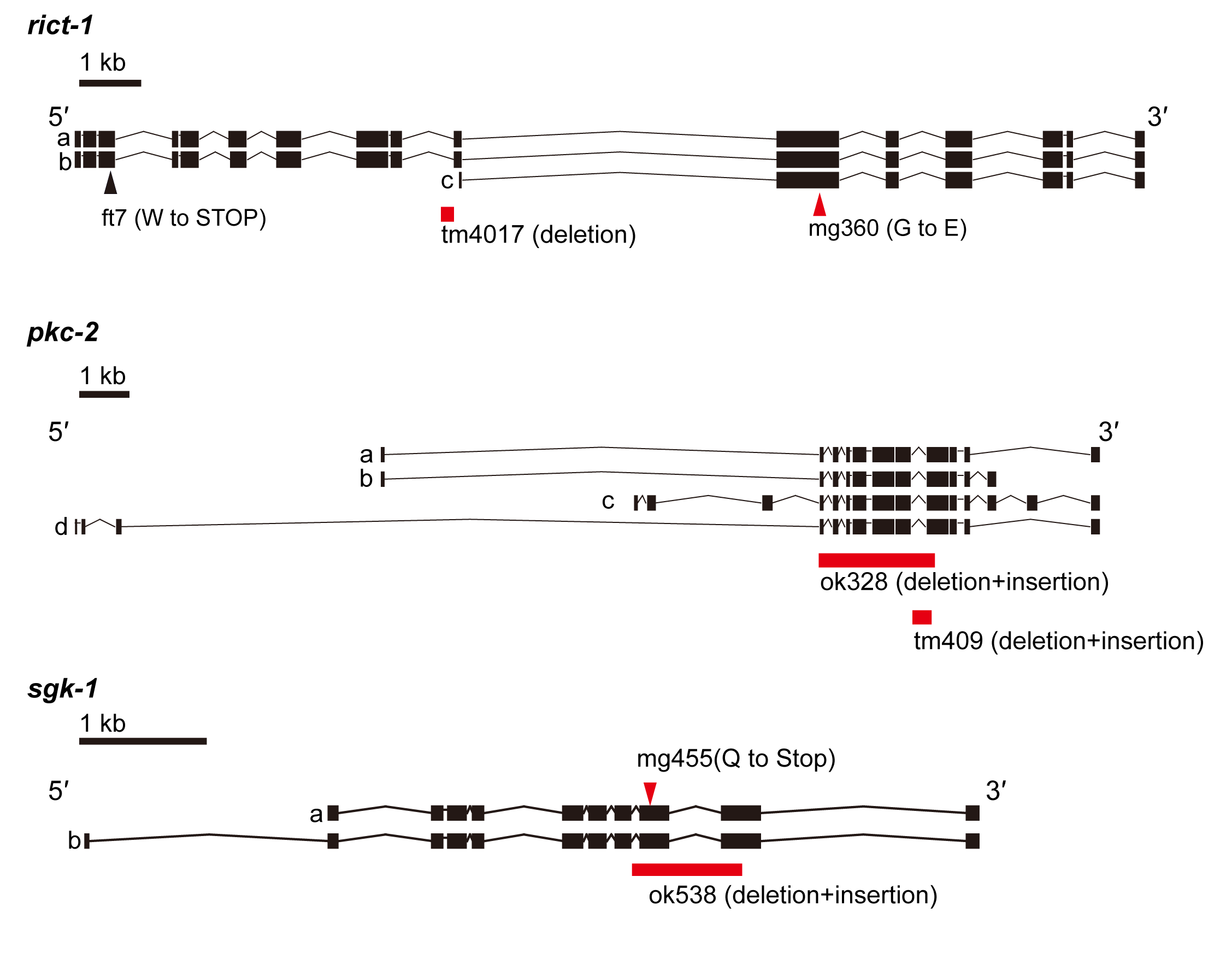

Supplement: S3 Fig — Genomic structure of rict-1 (top), pkc-2 (middle), and sgk-1 (bottom). Black boxes indicate exons, red boxes indicate deletion mutations, and arrowheads indicate point mutations. (TIF) [file pone.0177900.s003.tif]

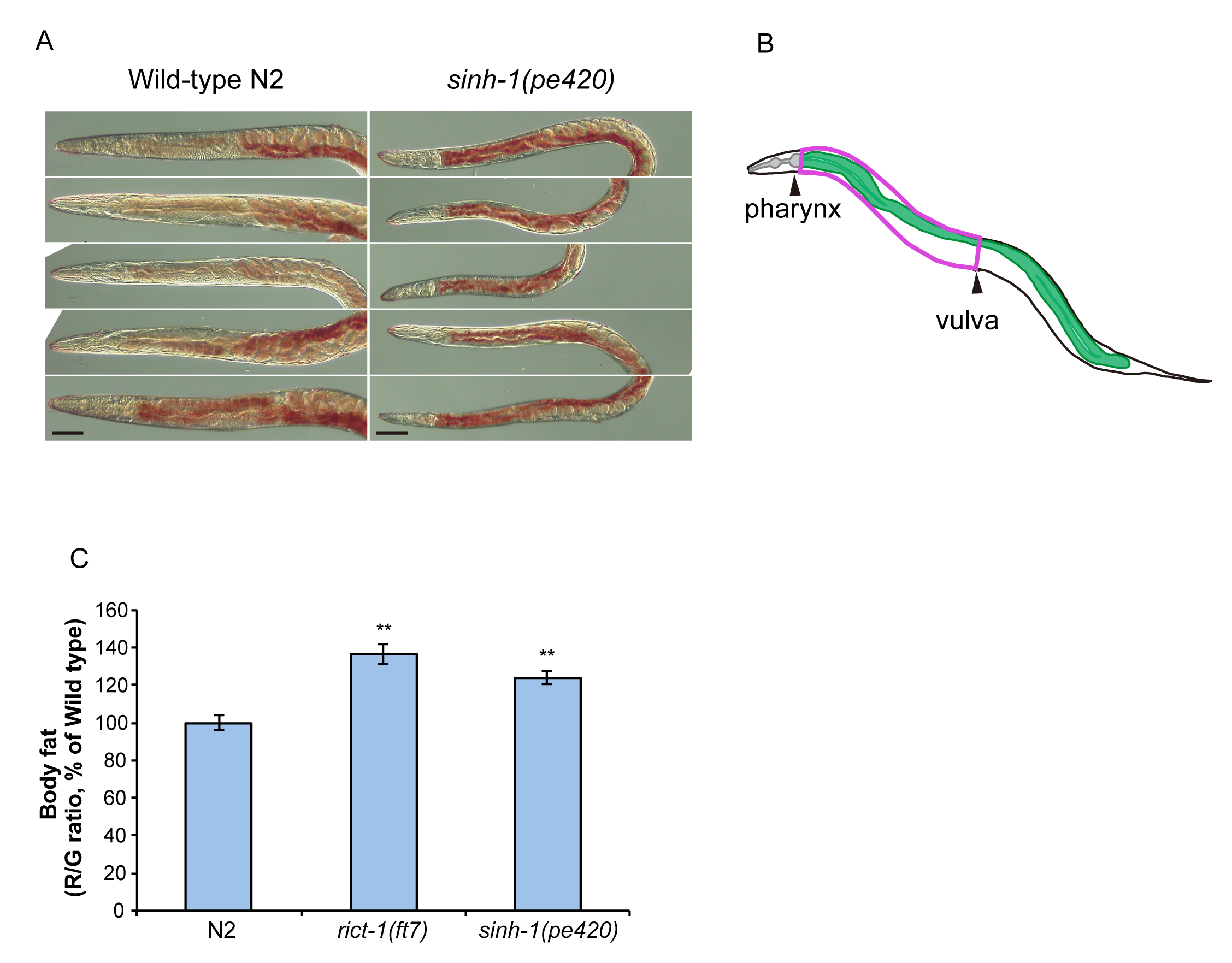

Supplement: S4 Fig — (A) Examples of Oil-Red-O-stained animals. Scale bars: 100 μm. (B) To quantify Oil-Red-O staining, we determined the R/G ratios of the region of interest, which included most of the anterior portion of the body: from the posterior edge of the pharynx to the vulva in each animal (indicated with a purple box). The details are listed in Materials and Methods. (C) Quantification of Oil-Red-O staining in wild-type, rict-1(ft7), and sinh-1(pe420) animals. As previously reported based on experiments conducted using Nile-Red staining [9], our Oil-Red-O staining results demonstrated a body fat increase in rict-1(ft7) animals. Similar to the rict-1(ft7) animals, sinh-1(pe420) animals also showed the increased body fat phenotype, which is in agreement with previous reports that Sin1 is an essential component of TORC2 in several species [22–24]. Data were normalized against the averaged value determined for wild-type. Error bars, s.e.m.; **p < 0.01 (wild-type vs. each mutant, Dunnett’s test, N = 40). (TIF) [file pone.0177900.s004.tif]

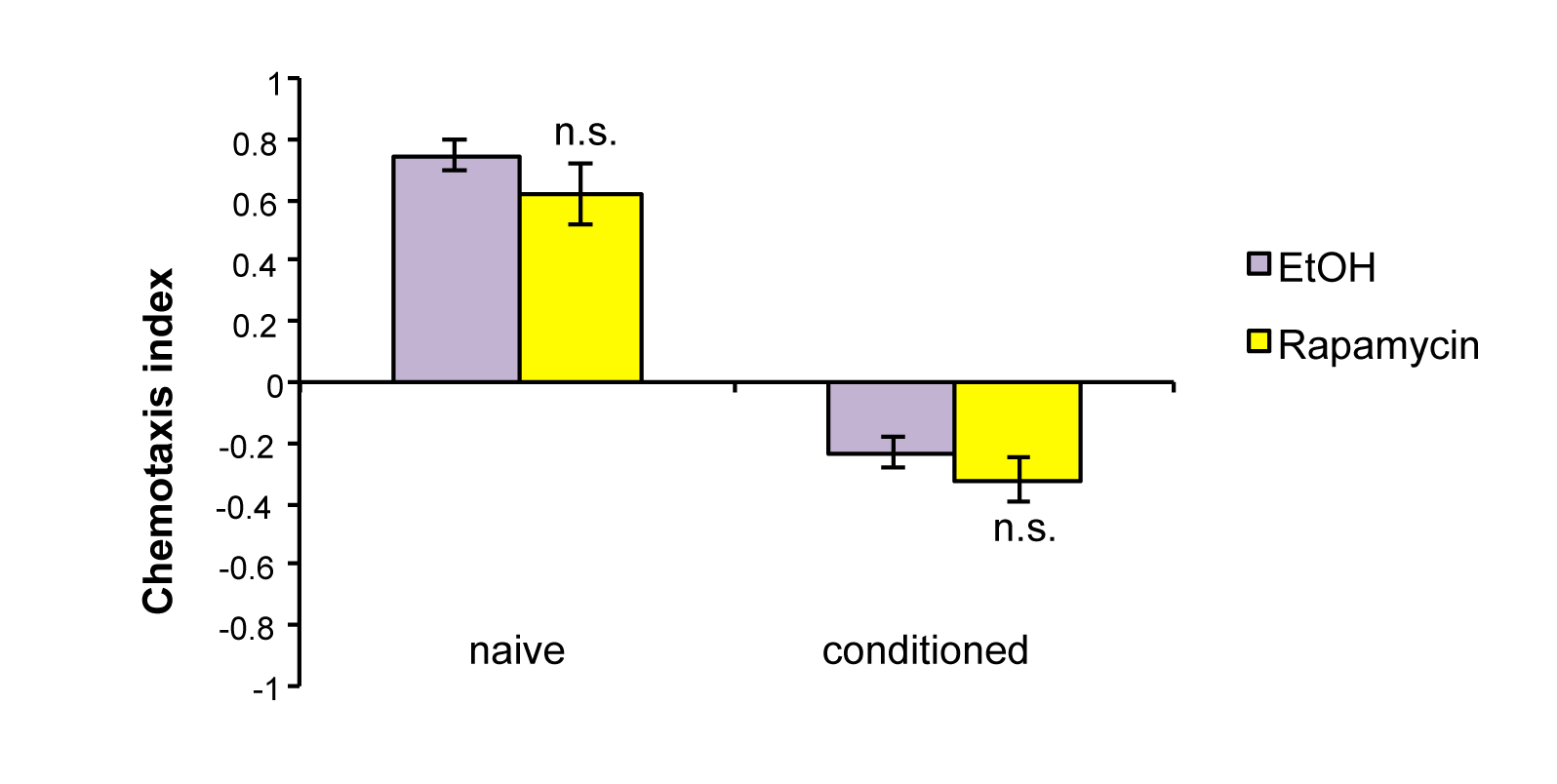

Supplement: S5 Fig — Rapamycin administration didn’t affect food-odor associative learning. This is consistent with the phenotype of rsks-1(ok1255) mutants, which showed no defect in food-odor associative learning. The learning assay was performed using benzaldehyde as an odorant as described in Fig 4F. Error bars, s.e.m.; n.s. = not significant (Student’s t test, N ≥ 8). (TIF) [file pone.0177900.s005.tif]

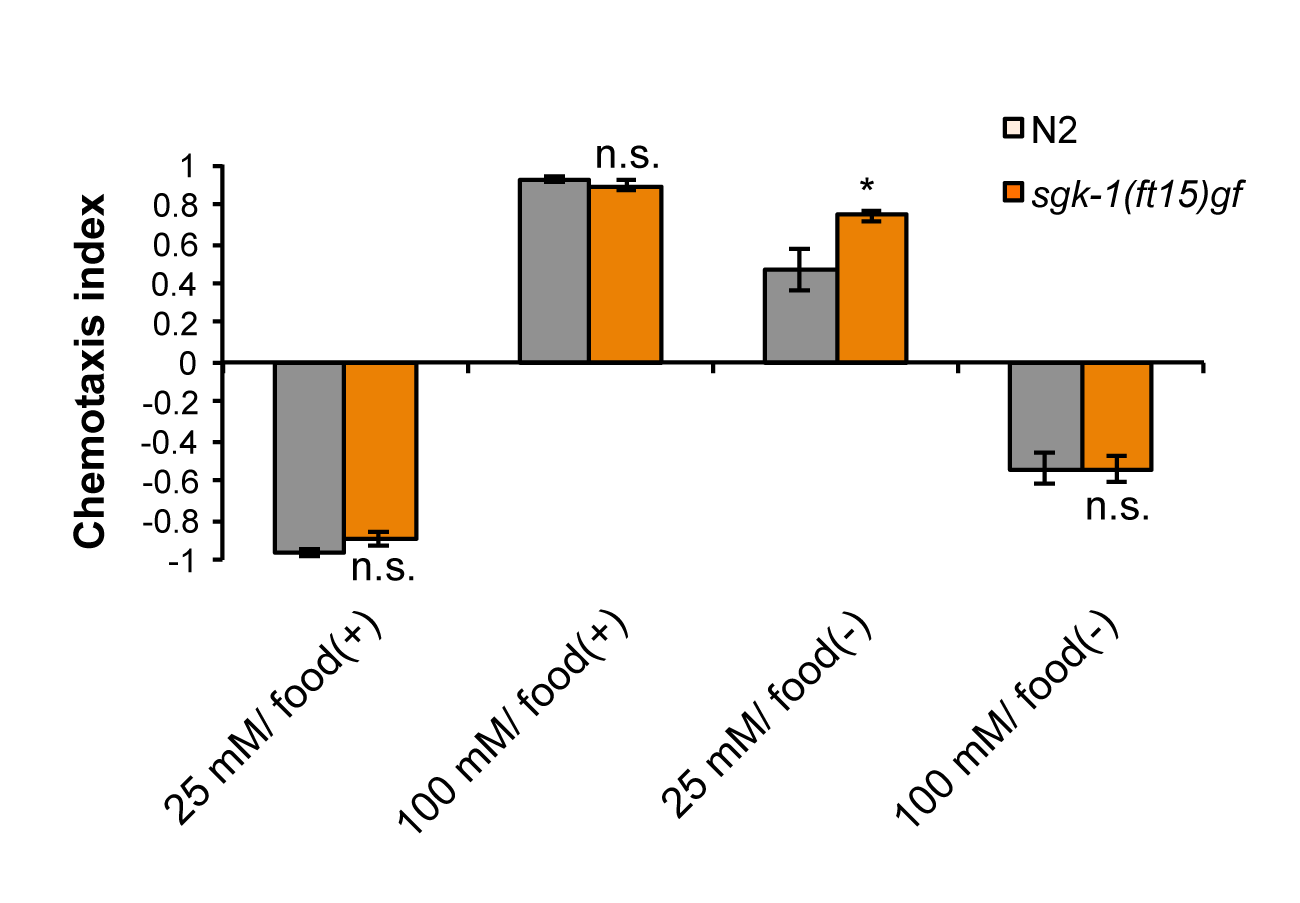

Supplement: S6 Fig — Chemotaxis of the sgk-1(gf) mutant after salt conditioning is shown. Error bars, s.e.m.; *p < 0.05, n.s. = not significant (Student’s t test, N ≥ 6). (TIF) [file pone.0177900.s006.tif]

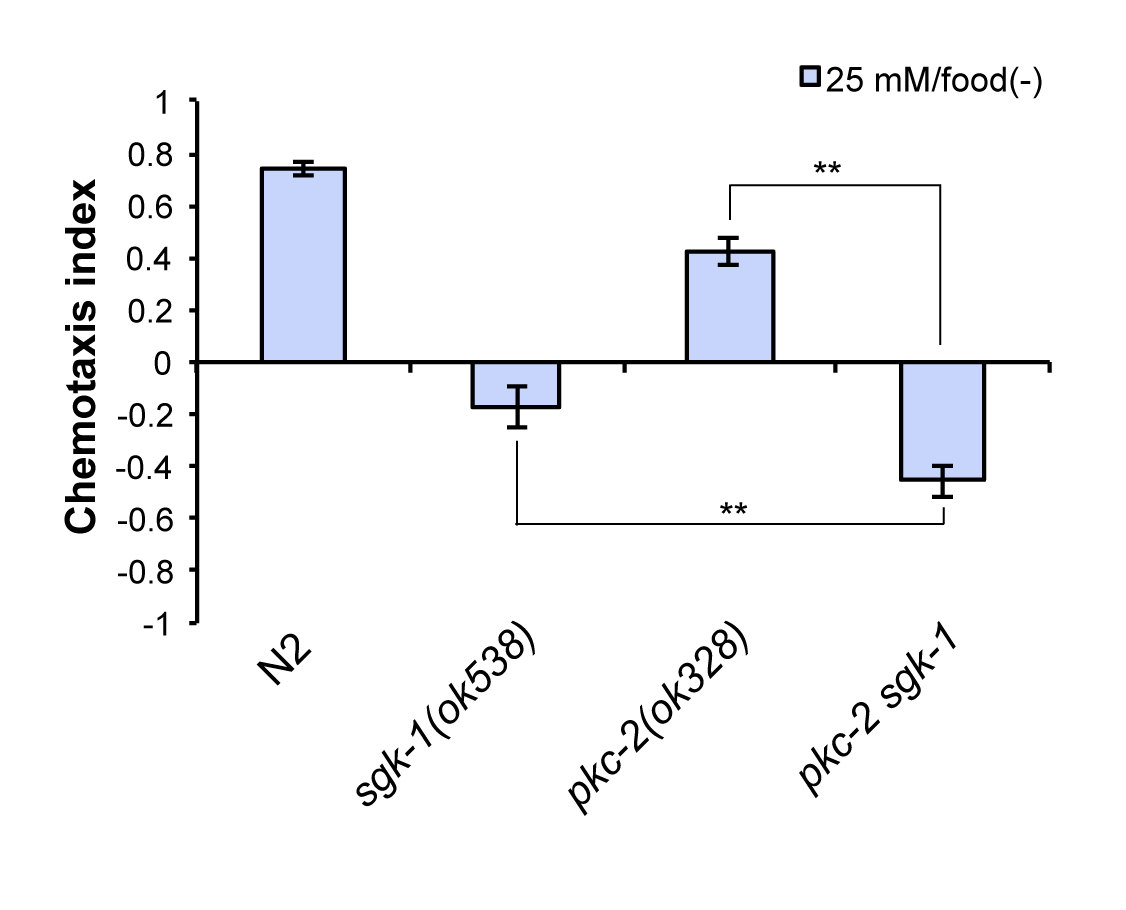

Supplement: S7 Fig — sgk-1;pkc-2 double mutants showed enhanced migration to low salt levels as compared with each single mutant. Error bars, s.e.m.; **p < 0.01 (Dunnett’s test, N ≥ 9). (TIF) [file pone.0177900.s007.tif]

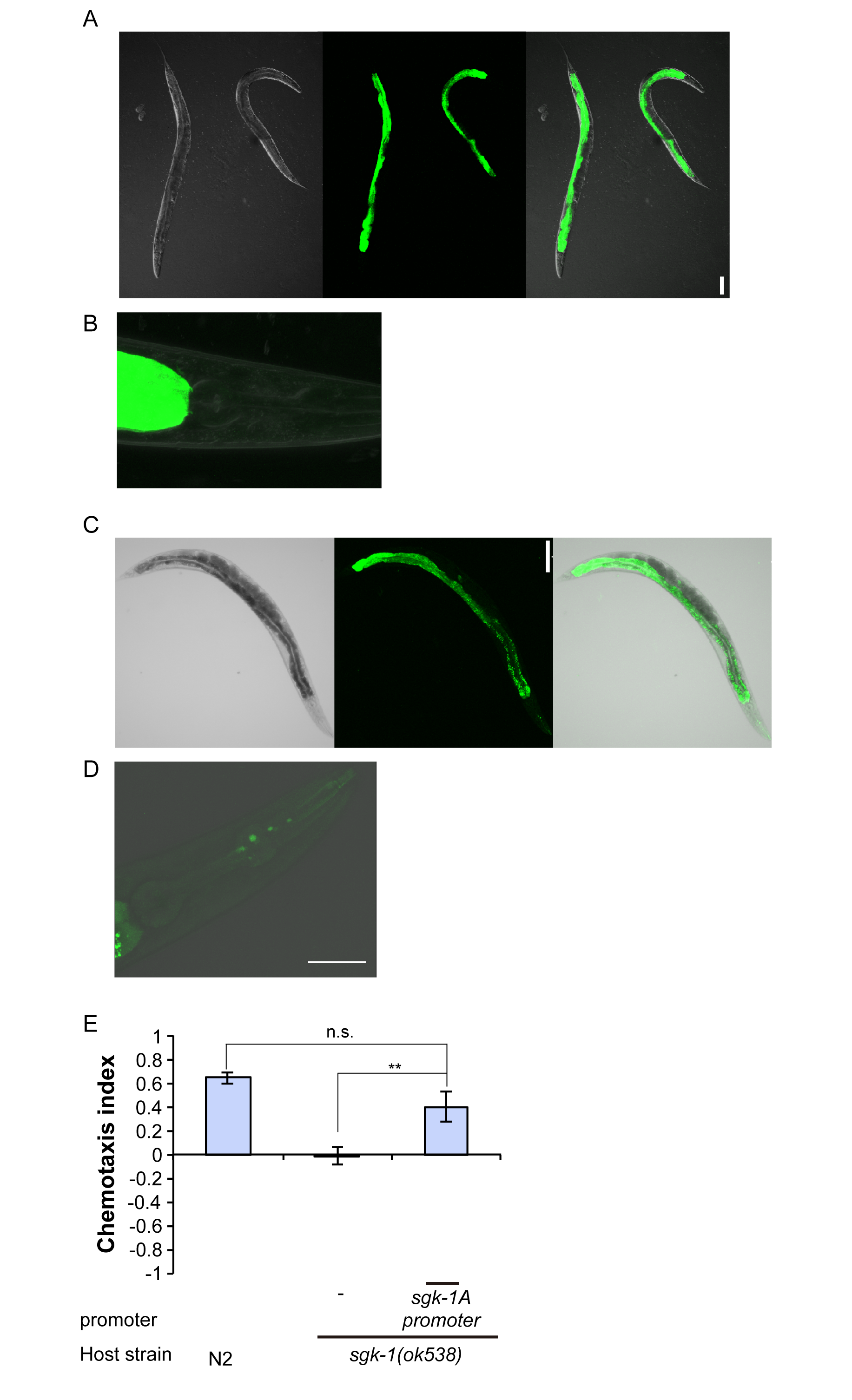

Supplement: S8 Fig — (A) Venus expression driven by the sgk-1 isoform A promoter (2.0 kb upstream of the first exon of sgk-1 isoform A). The sgk-1 isoform A promoter drives strong expression in the intestine, but not in neurons. Left, bright field; center, Venus; right, merged. Scale bar: 100 μm. (B) No signal was detected in the head ganglia. Scale bar: 30 μm. (C) Venus expression driven by the sgk-1 isoform B promoter (2.5 kb upstream of the first exon of sgk-1 isoform B). The sgk-1 isoform B promoter drives expression in the intestine. Scale bar: 100 μm. (D) No fluorescence was detected in the head ganglia. Scale bar: 30 μm. (E) Intestine-specific expression (sgk-1A promoter) of sgk-1 isoform A was sufficient for rescuing the chemotaxis defect of the sgk-1(ok538) mutant after low-salt/food(–) conditioning. Error bars, s.e.m.; **p < 0.01 (Dunnett’s test, N ≥ 9). (TIF) [file pone.0177900.s008.tif]

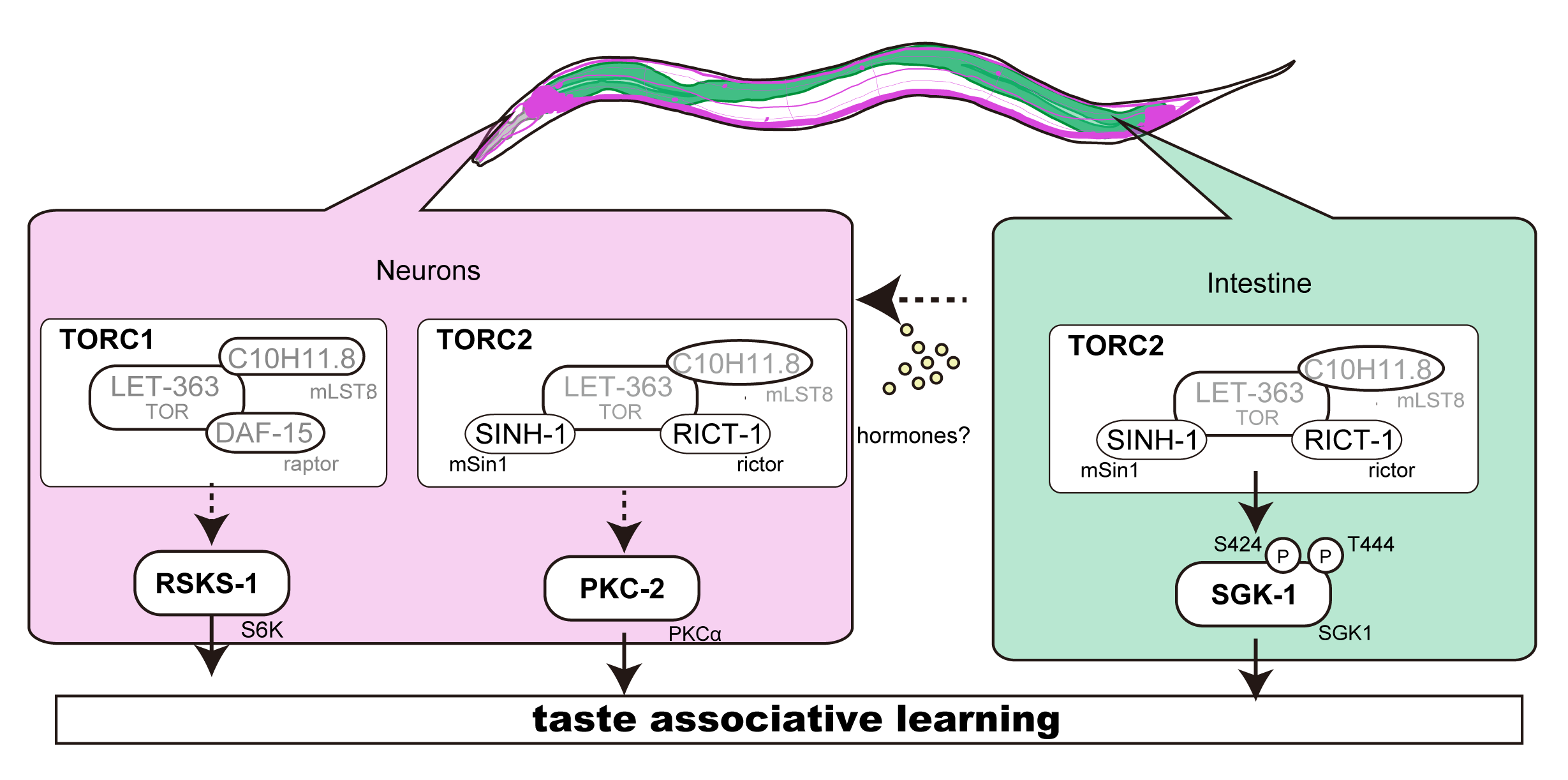

Supplement: S9 Fig — Intestinal TORC2/SGK-1 signaling and neuronal RSKS-1 and PKC-2 contribute to taste associative learning: TORC2/SGK-1 promotes migration to high salt levels through SGK-1 phosphorylation in the intestine after fed and starvation conditioning. (TIF) [file pone.0177900.s009.tif]

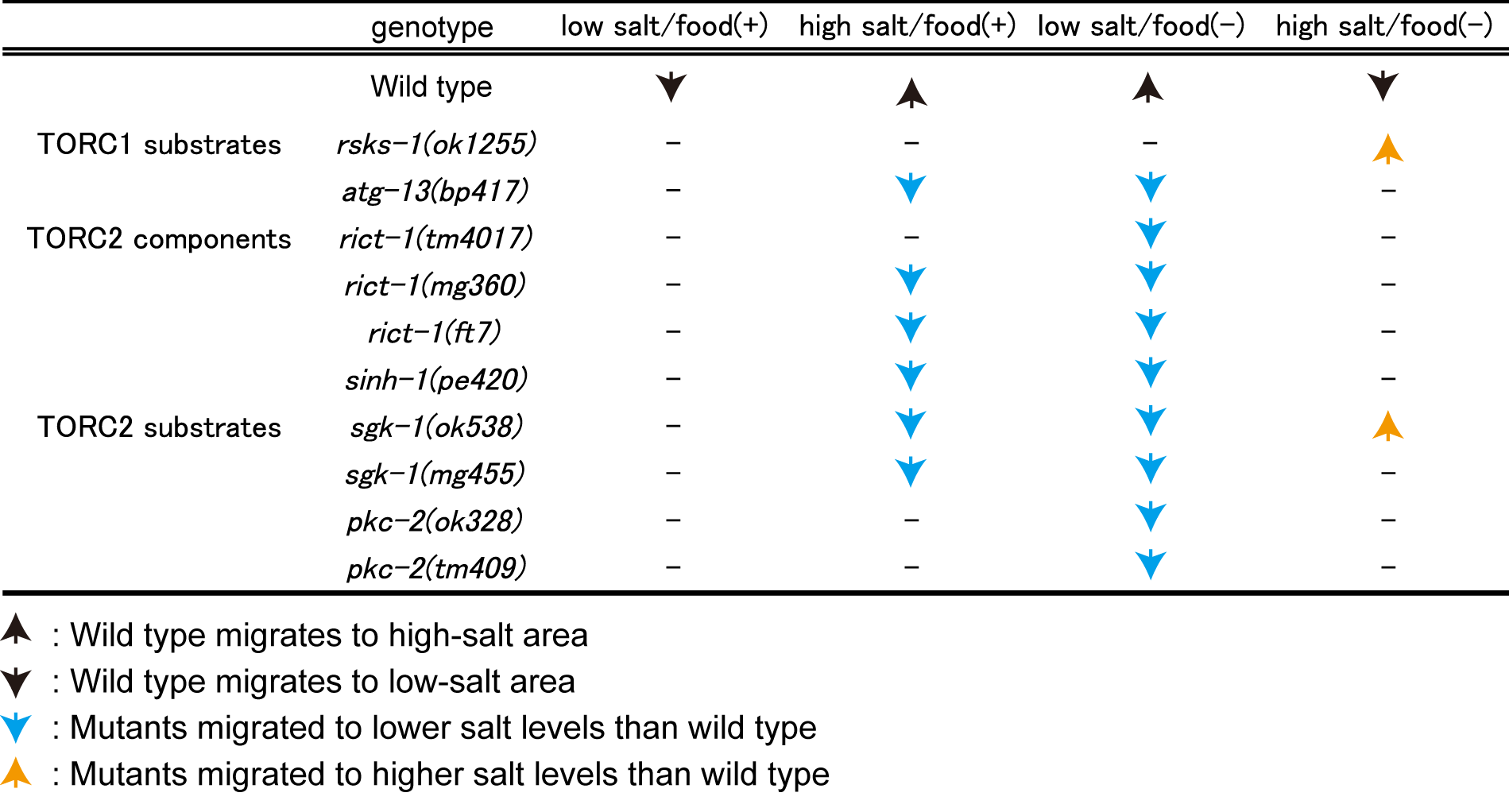

Supplement: S1 Table — (TIF) [file pone.0177900.s010.tif]
